# Gas Leakage Source Detection for Li-Ion Batteries by Distributed Sensor Array †

**DOI:** 10.3390/s19132900

**Published:** 2019-06-30

**Authors:** Valentin Mateev, Iliana Marinova, Zhelyazko Kartunov

**Affiliations:** 1 Department of Electrical Apparatus, Technical University of Sofia, Sofia 1797, Bulgaria; 2 HTS Design Department, Sensata Technologies Inc., Sofia 1582, Bulgaria

**Keywords:** catalytic gas sensors, impedance gas sensors, CO gas sensors, Li-Ion batteries, leakage source reconstruction, reconstruction algorithm

## Abstract

Lithium-based batteries operation is related to some safety risks of dangerous flaming, integrity destruction, or even explosion. Gas leakage is an early and reliable indicator for such irreversible malfunctioning of electrical accumulators. Often, accurate gas emission source location sensing is difficult especially in heavy operational conditions, related to temperature changes, vibrations, movements, accelerations, etc. In this paper we propose a gas detection system, with catalytic type sensor array, and a numerical reconstruction method for precise gas emission source location inside the battery pack. The detection system employs a distributed array of CO sensors. Proposed sensor array configurations significantly reduce the number of sensing nodes inside the battery pack and fewer sensors than the protected battery elements are used. This way, data acquisition process by sensor nodes is also simplified. Several array configurations are considered according to their measurement efficiency and accuracy. Reconstruction algorithm is based on fast interpolation technique very suitable for real-time data processing. Estimation of reconstruction method accuracy is made by computational model of the gas diffusion inside the pack.

## 1. Introduction

Electrochemical accumulator batteries are critical for modern technologies for transportation, computation, communication, and many more. At present, Li-Ion batteries are characterized by optimal energy and power density, long operational life, high efficiency in static and dynamic modes, etc. Nevertheless, these batteries could become unstable in certain conditions, causing dangerous flaming and explosion. Because of that, their operational conditions are continually monitored by precise protective controllers and sensors to overcome these issues. Monitoring systems employ mainly fast rising of temperature [1], pressure distributions changes [2], gas leakage caused by galvanic cell chemical degradation [3], or battery cell electric impedance changes [1,2,3,4]. Protective systems must react simultaneously in case of detected overheating, overcharging, short circuits, and even sudden impact [5]. Accumulator batteries for electric and hybrid vehicles are composed of thousands, even tens of thousands, of galvanic elements. Direct monitoring of each element is extremely difficult, due to the huge quantity of sensors required, multiple sensors wiring complexity, complicated signal routing, multiplexing of data channels, accurate and fast measuring, data processing, and protective system reaction. All together, these difficulties result in high cost of monitoring protective systems, reducing their field of applications [1,2,3,4,5]. 

Gas emissions from batteries give integral information about ongoing processes during operation. Electrolyte, organic insulation, and supplemental construction materials produce a complex mixture of gases [4]. These emissions could be related to battery operational modes where most intensive emissions are temperature dependent and therefore appear mostly in heavy charging modes, overload discharging, and different stages of thermal destruction, all related to significant and fast overheating. In closed domains, such as battery enclosures, these emissions are difficult to associate with their spatial origin, especially at low concentration levels. At those terms, the limit of detection (LoD) theory is significantly important to define true values especially at distant measurement points. According to the International Union of Pure and Applied Chemistry (IUPAC), the LoD is defined as the “smallest measure that can be detected with reasonable certainty for a given analytical procedure” [6]. As a statistical measure based on the standard deviation of a linear static output characteristic, the LoD could be extended for dynamic sensor response uncertainty [7] and multisensory measuring system calibration [8]. Numerical reconstruction methods for source location are well known approaches [7,8,9,10] but the computational complexity of inverse source reconstruction algorithms could be inappropriate for onboard monitoring system controllers [1]. Some reduced computational complexity methods, like artificial neural networks, are proposed in [8]. Another possibility for speeding up the reconstruction algorithms is by reducing the number of sensing nodes at acceptable uncertainty level [10,11,12,13].

In this paper are proposed a gas detection system with catalytic type sensor array and a reconstruction method for precise gas emission source location inside battery pack. Gas leakage is an early and reliable indicator for irreversible malfunctioning. This method is characterized by excellent gas source location selectivity, reduced number of sensors, and therefore high measuring process efficiency. 

The paper is organized as follows: (1) Introduction about protected battery pack module and consideration about selection of indicating gas is given (2); (3) technical description of used sensors, sensor array configurations, and consideration of their operational conditions are provided; (4) gas diffusion process is described by computational time-dependent model; (5) results about gas diffusion in different emission source cases is presented; results on sensor arrays performance are acquired also; (6) gas source reconstruction method is proposed in two variations, with or without considering gas diffusion time-constant. Actual reconstruction results based on predicted sensor readings are shown; (7) finally, conclusion on reconstruction method and results are given.

## 2. Battery Pack Module

**Figure 1:**
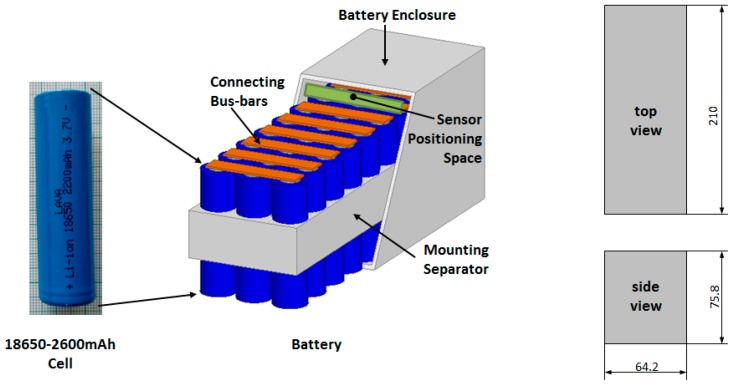
Battery pack module 30-ty pcs. of 18,650 cells and enclosure outer size. Sensor array is positioned on the top part of the enclosure, over the connection terminals.

The proposed automatic gas detection system is optimized for a battery pack module like that shown in Figure 1. The battery pack module is assembled by 30-ty 18650 Lithium-Ion Cells, with standard capacity (C) of 2600 mAh at nominal voltage 3.7 V [14]. The single battery cell is a cylinder with outer diameter of 18.6 mm by 65.2 mm length. The 18,650 cells have a standard discharge current of 0.2 C to a maximum of 1.0 C and can handle about 500 charging cycles. 

Entire battery pack dimensions without connection busbars and terminals, are 60.2 mm × 206 mm × 65.8 mm. The battery pack module is placed in a hermetic outer enclosure (Figure 1), with dimensions as follows: 64.2 mm × 210 mm × 75.8 mm. Sensors are placed inside the enclosure, over the connecting busbars. 

The electrolyte in a lithium-ion battery is flammable and generally contains lithium hexafluorophosphate (LiPF_6_) or other Li-salts, containing fluorine. In the event of overheating, the electrolyte evaporates and eventually will be vented out from the battery cells. The gases may ignite immediately, raising the risk for a gas explosion. Li-ion batteries release a various number of toxic substances as well as e.g., CO (an asphyxiant gas) and CO_2_ (induces anoxia) during heating and fire. At increased temperatures, the fluorine content of the electrolyte and other organic materials of the battery such as the polyvinylidene fluoride (PVdF) binder in the electrodes, may form gases such as hydrogen fluoride HF, phosphorus pentafluoride (PF_5_), and phosphoryl fluoride (POF_3_) [4]. 

**Figure 2:**
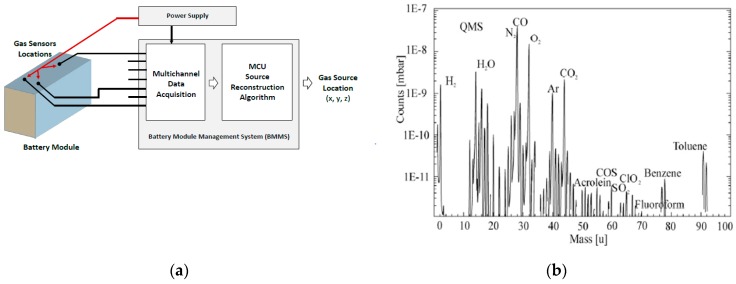
Battery management system block diagram (**a**); quadrupole mass spectrometry (QMS) analysis of lithium-ion battery destructive gas leakage (**b**) [4].

Quadrupole mass spectrometry (QMS) analysis of lithium-ion battery gas leakage content during destructive oxidation is shown in Figure 2b [4]. As a direct reliable indicator, the concentration of CO and CO_2_ is selected. Their concentrations could be easily observed over the natural existing levels of these gases. For CO, natural concentration is assumed to be below 9 ppm (10.48 mg/m^3^) and for CO_2_ it is around 400 ppm (465 mg/m^3^). Increased CO emission indicates initial temperature rising, while the presence of higher concentration levels of CO_2_ is a strong clue for intensive destructive oxidation. Both together could be combined for advanced sensitivity and selectivity of battery fault recognition. 

## 3. Gas Sensors 

In the proposed gas detection system are considered two types of CO sensors. The first one is thermal catalytic and the second one is diffusion impedance sensor. They are characterized by different static and dynamic range, accuracy. and sensitivity. Influence of these parameters over the measurement and reconstruction process is provided in the conclusion section of the paper. 

**Figure 3:**
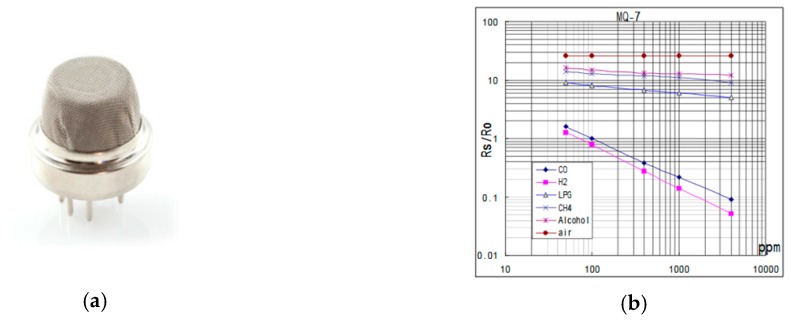
MQ-7 sensor outlook (**a**); and output MQ-7 sensor characteristics (**b**) [16].

The used gas catalytic type sensor is MQ-7 [15]. The internal circuit and design structure of MQ-7 gas sensor is shown in Figure 3. The sensor consists of two elements: A detector element, which contains catalytic material sensitive to the detected gases, and a reference compensator element, which is inert. Detected gases will burn only on the sensitive element, causing a rise in temperature and, as a consequence, a rise in its electrical resistance. Detected gases will not burn on the compensator—its temperature and resistance will remain unchanged. 

**Figure 4:**
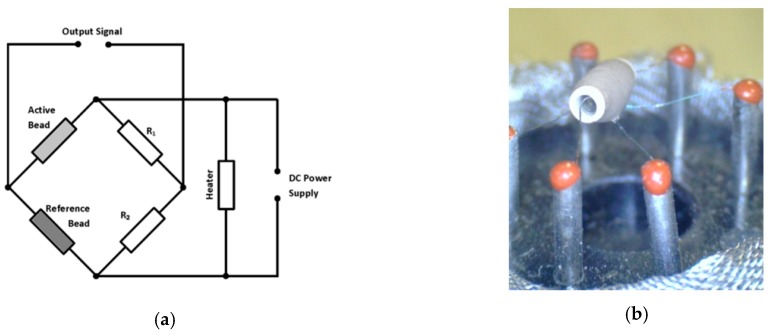
MQ-7 sensor bridge wiring diagram (**a**); heating bed and sensing electrode (**b**).

The sensor is composed of a micro Al_2_O_3_ ceramic tube, Tin Dioxide (SnO_2_) sensitive layer, and a measuring electrode and heater are fixed into a crust made by a plastic and stainless-steel net (Figure 3a and Figure 4b). The heater provides necessary operational conditions for the sensitive components. The enveloped MQ-7 has six pins; four of them are used for signals acquisition and the other two are used for providing heating current (Figure 4a). 

Normally, a Wheatstone bridge circuit is formed with the sensor elements as it is shown in Figure 4a. A heater is adjusted to maintain a state of balance of the bridge circuit in clean air, free of combustible gases. 

The measured gas concentration will affect the detector element resistance (active bead), which will rapidly rise, causing an imbalance in the bridge circuit, thus producing an output voltage signal. The output voltage signal is proportional to the concentration of the gas as it is shown in Figure 3b. Gas concentration can be determined by output voltage measurements. 

Recently. A new generation of electrical impedance gas sensing platforms was announced. They are characterized by increased sensitivity, compact size, and reduced energy consumption. Electrochemical sensor platform BE3 [17] is designed for different gases detection. It is a three-electrode structure with working, reference, and counter electrodes. Patterns of reference, counter, and working electrodes are created by sputtering of platinum on a ceramic substrate. The sensitive layer on the platform can be applied by various thick- or thin-film technologies [15]. 

Dimensions: 7.25 mm × 8.75 mm × 0.6 mm, Detection of H, CO_2_, NO_2_ gases, with functional layer of solid polymer electrolyte, with linear sensitivity of 600 nA/ppm, in detection range of 0–10 ppm. 

**Figure 5:**
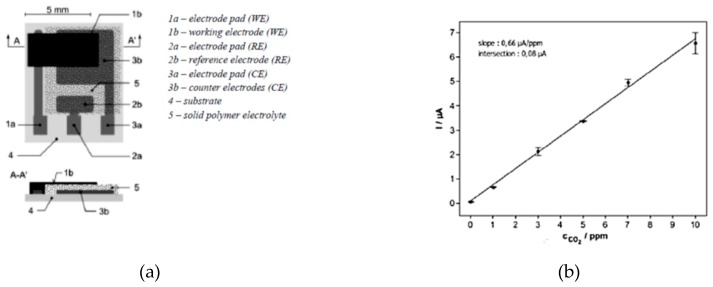
Sensor layered design (**a**); and output characteristic of the BE3 sensor platform (**b**) [15].

MQ-7 sensor LoD after calibration for the particular implementation is found to be 30 ppm (35 mg/m^3^ CO) (Figure 5) [15]. Sensors’ dynamic characteristics: MQ-7 sensor response-time (dynamic resistance change to gas concentration) is 60 s. For BE3, response time is in the 60/100 s range [15,17].

The heater of the MQ-7 sensor significantly increases the energy consumption compared to BE3. Because of that, MQ-7 sensors are set up to operate only if battery current charging/discharging is greater than 0.1 C or 260 mA per battery element [16]; also, sensor operation covers the 15 min period after the electric current consumption is interrupted. According to the measurements, charging and discharging intervals are most risky to initiate destructive reaction associated with gas emission [18,19]. 

## 4. Gas Diffusion 

**Figure 6:**
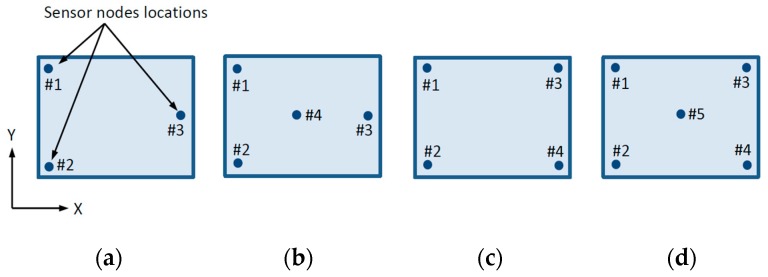
Sensor array nodes configurations are as follows: Three-node configuration (**a**), four-node configuration (**b**), four-corner-node configuration (**c**), and five-node configuration (**d**).

Gas diffusion distribution inside the battery pack module is rather complex because of small, specifically shaped elements in the battery enclosure, temperature, and pressure variations. Complex gas distribution makes gas source location extremely difficult especially in transient processes. Here, a gas diffusion model is used for reconstruction method calibration and selection of optimal sensor array configuration. Four array configurations are considered (Figure 6), with three, four, and five sensing nodes. 

A computational model for CO and CO_2_ gas diffusion in battery pack module, of which sizes are shown in Figure 1, is formulated. The model considers a single source of gas emission i.e., single battery element. 

Diffusion of two component gas mixture in closed domain is described by Equation (1)

(1) $$\frac{\partial c}{\partial t}=D\nabla^{2}c-v\nabla c,$$

where *c* is the gas concentration, *D* is the diffusion coefficient constant, **v** is the distribution velocity vector, $\nabla^{2}$ is the Laplacian. The CO_2_ diffusion coefficient in air, at 22 °C, 101.32 kPa, is 0.14 cm^2^/s. CO diffusion coefficient at same conditions is 0.19 cm^2^/s. 

Time-dependent finite element method (FEM) formulation for gas diffusion distribution modeling is used and implementation is made by Ansys software package [20]. Concentrations are taken in specific measuring points, corresponding to four sensor positioning patterns (Figure 6). 

Temperature and pressure change effects are neglected in the modeling process. 

Four sensor array arrangements are introduced (Figure 6). Sensors are placed on the battery’s top layer, inside the module outer enclosure and above the main connection bus bars. Figure 6 is a schematic representation of a closed air volume with gauge size of battery internal volume where gas diffusion is measured on the top surface. Corner nodes are located on 20 mm from horizontal enclosure edges to avoid wall proximity effects; center nodes on Figure 6b,d are located in the horizontal center of the battery enclosure. 

## 5. Diffusion Modeling Results

**Figure 7:**
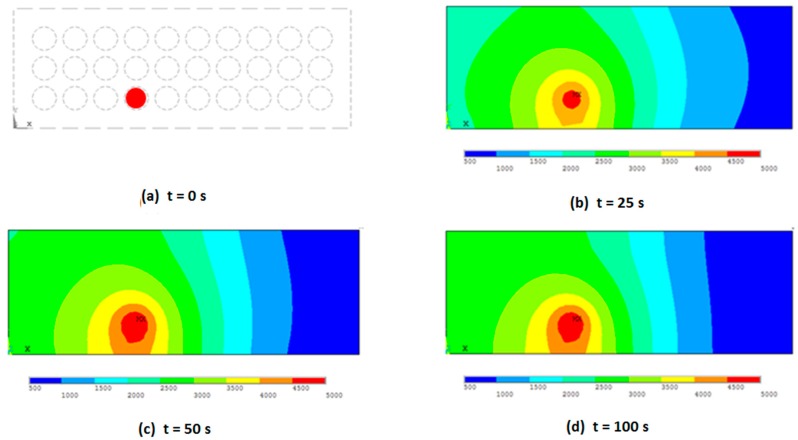
Transient gas distribution results for 5000 ppm (5823 mg/m^3^) in 100 s range. Gas emission source is marked in red in t = 0 (**a**), concentrations for time intervals t = 25 s, t = 50 s, and t = 100 s are shown in (**b**), (**c**) and (**d**) respectively.

Time-dependent simulation results for CO gas leakage distribution are obtained and are shown in Figure 7. Gas source is marked in red in Figure 7a; it is placed in the middle of the pack, where circles are battery elements in top view. Tested source concentration is 5000 ppm (5823 mg/m^3^) for 100 s time interval with 0.25 s time step. 

**Figure 8:**
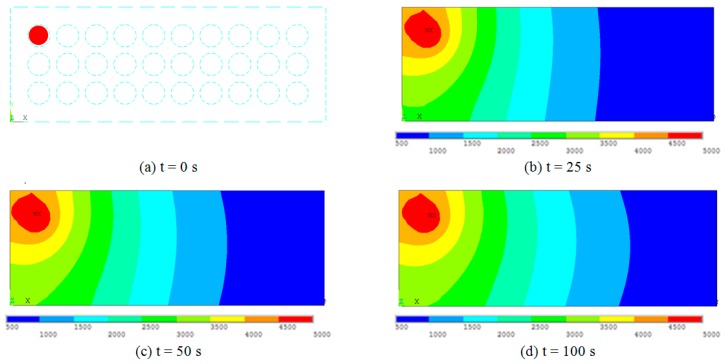
Transient gas distribution results for 5000 ppm (5823 mg/m^3^) in 100 s range. Gas emission source is marked in red t = 0 (**a**) concentrations for time intervals t = 25 s, t = 50 s, and t = 100 s are shown in (**b**), (**c**) and (**d**) respectively.

The second results dataset for different gas emission source location are shown in Figure 8. Gas source is marked in red in Figure 8a; it is placed close to the edge of the pack. In both modeled cases, the CO gas concentration rises over the time, starting from source element and approaching the steady-state values at the end of the modeling time-period. Proximity of domain edges is important for gas distribution pattern. 

**Figure 9:**
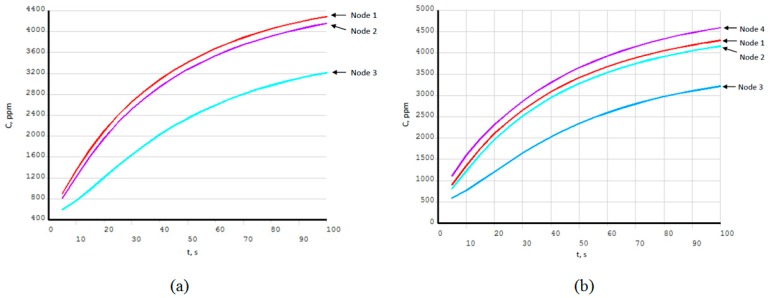

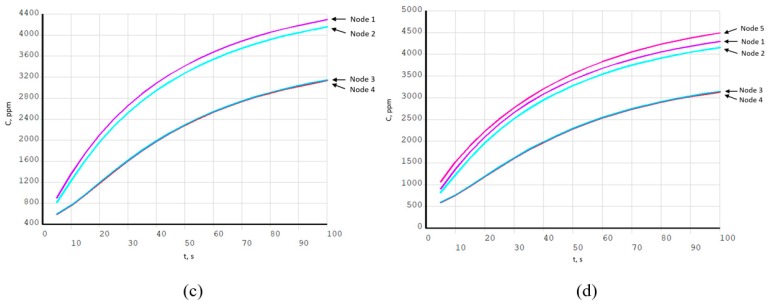
Transient gas concentrations for three-node (**a**), four-node (**b**), four-corner-node (**c**), and five-node (**d**) configurations with 5000 ppm (5823 mg/m^3^) source; gas source location is shown in Figure 7. These data are used for source reconstruction method.

**Figure 10:**
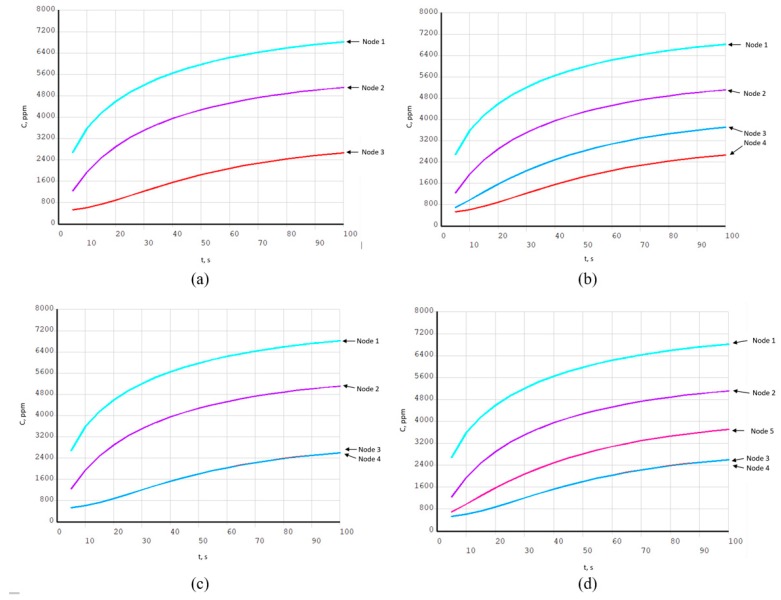
Transient gas concentrations for three-node (**a**), four-node (**b**), four-corner-node (**c**), and five-node (**d**) configurations with 10,000 ppm (11,646 mg/m^3^) source; gas source location is shown in Figure 8. These data are used for source reconstruction method.

Gas concentration values are taken at sensor array nodes positions, as they are described in Figure 6. These transient gas concentration changes in sensor nodes for four sensor array configurations are shown in Figure 9 and Figure 10. Concentration values are related to the distance to the gas source location and battery pack wall distance. These dynamic data are suitable for source location reconstruction method calibration and error estimation. 

Results shown in Figure 9 are corresponding to Figure 7 gas distributions where concentration values are taken at sensor nodes positions, introduced in Figure 6. Sensor array configurations are: Three nodes in Figure 9a, four nodes in Figure 9b, four corner nodes in Figure 9c, and five nodes in Figure 9d. 

Results shown in Figure 10 are corresponding to Figure 8 gas source location. Tested source is with 10,000 ppm (11,646 mg/m^3^) concentration for 100 s time interval at 0.25 s time step. Concentration values are taken at sensor nodes positions, where the sensor array configuration patterns are: Three nodes in Figure 10a, four nodes in Figure 10b, four corner nodes in Figure 10c, and five nodes in Figure 10d. Node numbering is according to Figure 6. 

These data are used for inverse gas source location reconstruction method calibration and uncertainty estimation. 

**Figure 11:**
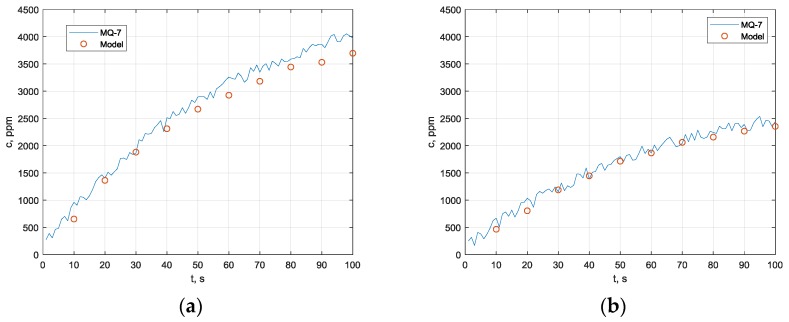
Concentration results for 50 mm distance to the sensor (**a**) and 100 mm distance (**b**). Red dots are expected concentration values calculated by diffusion model.

Experimental validation of modeled results is performed with the MQ-7 [15] sensor. For sensor calibration, digital carbon monoxide meter M0198108 is used; calibration is up to 1000 ppm concentration. Sensor output voltage drop signal is acquired by NI DAQ USB- 6009 data acquisition device and NI LabView virtual instrument. Measured results at two different distances are shown in Figure 11a,b (50 mm, 100 mm). Estimated uncertainty in comparison with the computational model is below 12% of maximum concentration level. 

## 6. Source Reconstruction Method

Gas source reconstruction method is based on linear interpolation where source location is calculated by measured gas concentrations at the sensor nodes. Four sensor nodes arrangements are introduced (Figure 6). Sensors are placed on the battery’s top layer, inside module outer enclosure, and above main connection bus bars. Figure 6 is a schematic representation of a closed air volume with gauge size of battery volume where gas diffusion is measured on the top surface. 

Reconstruction is performed on steady-state gas concentrations *C_∞_* where the expected value is calculated by Equation (2) using the moment local values at each of measurement nodes *C_i_(t)*,

(2) $$C_{i}{}_{\infty }=\frac{C_{i}(t)}{\left(1-e^{-\frac{t_{}}{T}}\right)},$$

where *t* is the local time for each reconstruction step and *T* is the time-constant formed by sensor response time, size, and difusion properties of closed gas volume. Empirical estimation of the time-constant taking two secuantional concentration values *C(t_2_)/C(t_1_)* for time interval *∆t = t_2_ − t_1_* is given by Equation (3).

(3) $$T=\frac{\Delta t}{\ell n\left(\frac{C(t_{2})}{C(t_{1})}-1\right)}$$

For the three-sensor configuration (Figure 6a), the gas concentration *C* at any point location denoted by two-dimensional Cartesian coordinate system (*x, y*) can be calculated as a function of measured gas concentrations at sensing nodes locations by Equation (4).

(4) $$C(x,y)=N_{1}C_{1}+N_{2}C_{2}+N_{3}C_{3},$$

where *C_i_* are steady-state gas concentrations at sensor nodes locations Equation (2) and *N_i_* are planar approximation basis functions, expressed by Equation (5)

(5) $$N_{i}=k(a_{i}+b_{i}+c_{i}y),$$

where *a_i_, b_i_,* and *c_i_* are linear coefficients for relative distance between measuring nodes [21]. 

Source location is calculated by finding the approximated maximal gas concentration, Equation (6),

(6) $$\max(C(x,y)=\sum_{i=1}^{n}N_{i}C_{i}),$$

where *x, y* values are the locations of all battery pack elements. Reconstruction algorithm sequence diagram is shown in Figure 9.

(7) $$C(x,y)=\sum_{i=1}^{n}N_{i}C_{i},$$

Concentration interpolation for higher node arrangements (Figure 6b–d) are expressed by Equation (7). 

**Figure 12:**
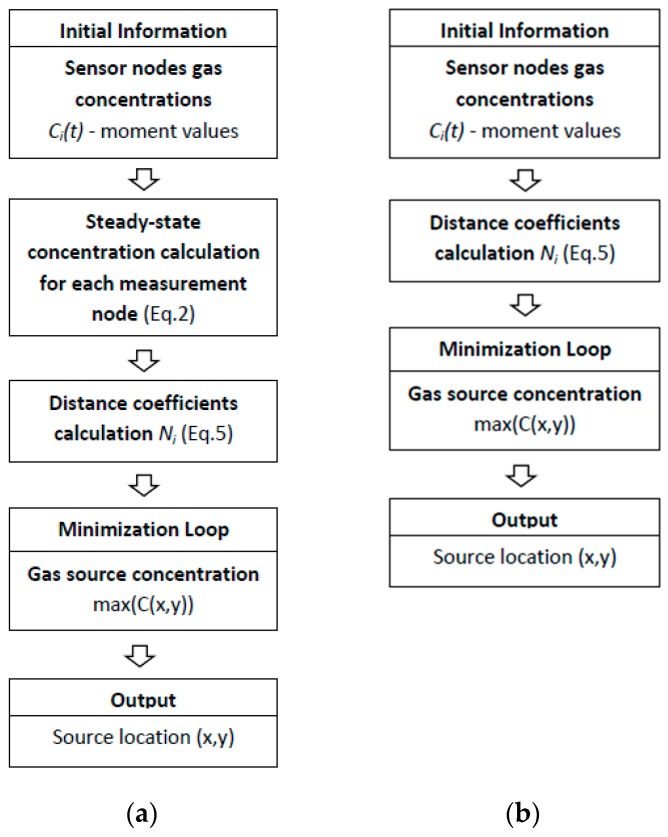
Reconstruction algorithms sequence diagram. Algorithm with steady-state concentration values (**a**) and with local concentration moment values (**b**). Second algorithm modification is with lower computational cost and little bit faster.

Reconstruction algorithm sequence diagram is shown in Figure 12. Two modifications are considered; the first one (Figure 12a) uses steady state gas concentration predicted by Equation (3). In the second one, modification is using currently measured gas values, registered by the sensors in real time (Figure 12b). 

Real-time gas source reconstruction implemented in the second method eliminates the computational heavy solutions of Equations (2) and (3). This simplification increases the number of devices capable to implement the algorithm while also reducing reconstruction method sensitivity especially in lower gas concentration levels. 

**Figure 13:**
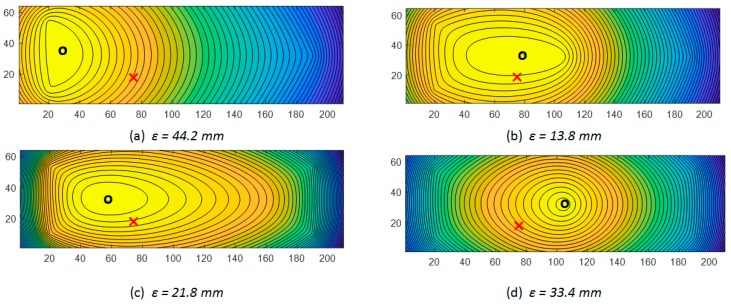
Reconstructed concentration maximums by steady-state concentration algorithm, concentrations for three-node (**a**), four-node (**b**), four-corner-node (**c**), and five-node (**d**) sensor array configurations. Red crosses represent the original known source location (Figure 7a); the black circles indicate reconstructed maximum concertation. For each node scheme, absolute distance between original and reconstructed source location—*ε* is calculated. Values of ε that are smaller than 18.6 mm are accurate enough to locate a single element as a source of gas emission.

Reconstructed concentration maximums for Figure 7 data are shown in Figure 13. Results are acquired by steady-state concentration algorithm, for three-node (a), four-node (b), four-corner-node (c), and five-node (d) sensor array configurations. For each node scheme, absolute distance *ε* between original (*x_s_, y_s_*) and reconstructed (*x_r_, y_r_*) source location coordinates – *ε* = $\sqrt{{\left(x_{s}-x_{r}\right)}^{2}+{\left(y_{s}-y_{r}\right)}^{2}}$ is calculated. Values smaller than 18.6 mm *ε* are accurate enough to locate a single element as a source of gas emission, where 18.6 mm is a single battery element diameter. 

**Figure 14:**
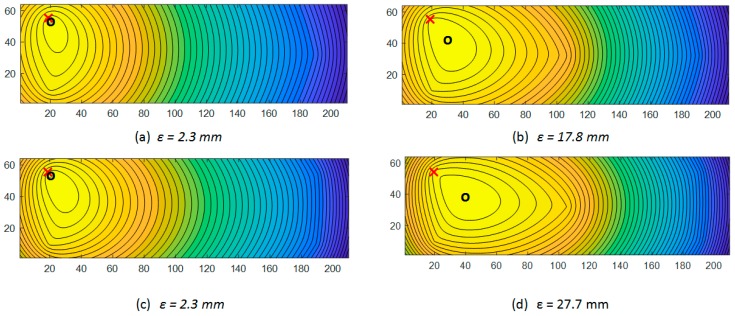
Reconstructed concentration maximums by steady-state concentration algorithm, concentrations for three-node (**a**), four-node (**b**), four-corner-node (**c**), and five-node (**d**) sensor array configurations. Red crosses represent the original known source location (Figure 8a); the black circles re point to reconstructed maximum concertation. For each node scheme, absolute distance between original and reconstructed source location—ε is calculated. Values of ε that are smaller than 18.6 mm are accurate enough to locate a single element as a source of gas emission.

Reconstructed concentration maximums for Figure 8 data are shown in Figure 14. Results are acquired by steady-state concentration algorithm, for each sensor array. Red crosses represent the original known source location (Figure 7a); the black circles point to reconstructed maximum concertation. 

**Figure 15:**
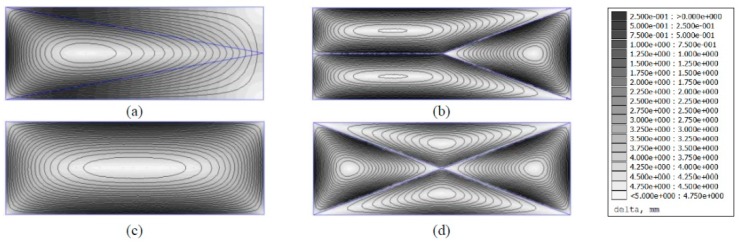
Uncertainty field distribution for three-node (**a**), four-node (**b**), four-corner-nodes (**c**), and five-node (**d**) sensor configurations. White zones represent maximal sensitivity acquired by relative proximity to sensor nodes.

Estimated optimal sensitivity fields for tested sensor configurations are shown in Figure 15. These results are based on catalytic gas sensor sensitivity; for accurate cell recognition, distance uncertainty value must be below 18.6 mm for the 18650 cell element. 

**Figure 16:**
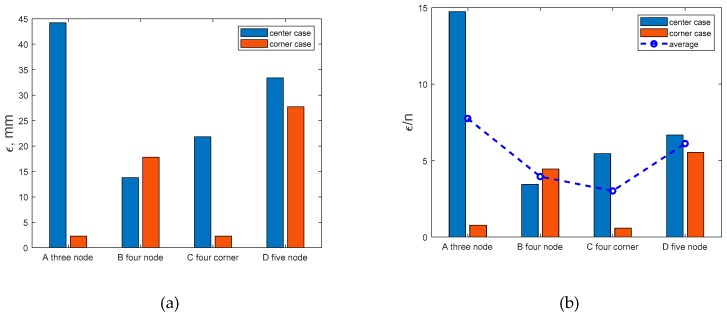
Uncertainty of reconstructed gas source location—ε (**a**), and uncertainty per measuring nodes number—ε/n (**b**). Blue bars correspond to central source case; red are for near corner located source case. Average uncertainty minimum is in four-node configurations.

Comparison of estimated absolute distance difference between original and reconstructed source location for each sensor array configuration is shown in Figure 16a. Reconstructed source location uncertainty per measuring nodes number (ε/n) is shown in Figure 16b. It is found that average uncertainty minimum is in four node configurations (Figure 16b, blue dotted line) There is a maximal reconstruction accuracy achieved by minimum number of sensing nodes. 

## 7. Conclusions

Proposed here is a gas detection system, with catalytic type sensor array, and a numerical reconstruction method for precise gas emission source location inside the battery pack has been successfully tested according to their accuracy. The detection system employs a distributed array of CO sensors where diffusive impedance sensors were found to be more suitable compared to catalytic ones for implementation in sensor arrays because of reduced energy consumption in continued use. Several sensor array configurations are considered according to their measurement efficiency and accuracy. Proposed sensor arrays configurations significantly reduce the number of sensing nodes inside the battery pack and used sensors are significantly less than the protected battery elements. Four-node and four-corner-node array configurations have been found to be the most accurate in the tested gas emission source locations. Their recognition distance uncertainty value is below the battery element size. Reduction of sensing nodes is related to data acquisition process and reconstruction method complexity, both of which are reduced in the four-node configuration. Reconstruction algorithm is based on fast interpolation technique very suitable for real-time data processing. Gas source reconstruction method is analyzed in two variations, with or without considering gas diffusion time-constant. Estimation of reconstruction method accuracy is made by the computational model of the gas diffusion inside the pack. The proposed methodology could be relatively easily implemented in the existing battery management systems.
